# Single-Point Incremental Forming of Two Biocompatible Polymers: An Insight into Their Thermal and Structural Properties

**DOI:** 10.3390/polym10040391

**Published:** 2018-04-01

**Authors:** Luis Marcelo Lozano-Sánchez, Isabel Bagudanch, Alan Osiris Sustaita, Jackeline Iturbe-Ek, Luis Ernesto Elizalde, Maria Luisa Garcia-Romeu, Alex Elías-Zúñiga

**Affiliations:** 1 Tecnologico de Monterrey, Escuela de Ingeniería y Ciencias, Ave. Eugenio Garza Sada 2501, Monterrey 64849, México; alan.sustaita@itesm.mx (A.O.S.); jackeline.iturbe.ek@itesm.mx (J.I.-E.); aelias@itesm.mx (A.E.-Z.); 2 Mechanical Engineering and Industrial Construction Department, Campus Montilivi, University of Girona, 17071 Girona, Spain; isabel.bagudanch@udg.edu (I.B.); mluisa.gromeu@udg.edu (M.L.G.-R.); 3 Centro de Investigación en Química Aplicada, Blvd. Ing. Enrique Reyna No. 140, Saltillo 25253, México; luis.elizalde@ciqa.edu.mx

**Keywords:** polycaprolactone, ultra-high molecular weight polyethylene, incremental forming, SPIF, XRD, chain orientation

## Abstract

Sheets of polycaprolactone (PCL) and ultra-high molecular weight polyethylene (UHMWPE) were fabricated and shaped by the Single-Point Incremental Forming process (SPIF). The performance of these biocompatible polymers in SPIF was assessed through the variation of four main parameters: the diameter of the forming tool, the spindle speed, the feed rate, and the step size based on a Box–Behnken design of experiments of four variables and three levels. The design of experiments allowed us to identify the parameters that most affect the forming of PCL and UHMWPE. The study was completed by means of a deep characterization of the thermal and structural properties of both polymers. These properties were correlated to the performance of the polymers observed in SPIF, and it was found that the polymer chains are oriented as a consequence of the SPIF processing. Moreover, by X-ray diffraction it was proved that polymer chains behave differently on each surface of the fabricated parts, since the chains on the surface in contact with the forming tool are oriented horizontally, while on the opposite surface they are oriented in the vertical direction. The unit cell of UHMWPE is distorted, passing from an orthorhombic cell to a monoclinic due to the slippage between crystallites. This slippage between crystallites was observed in both PCL and UHMWPE, and was identified as an alpha star thermal transition located in the rubbery region between the glass transition and the melting point of each polymer.

## 1. Introduction

**Figure 1:**
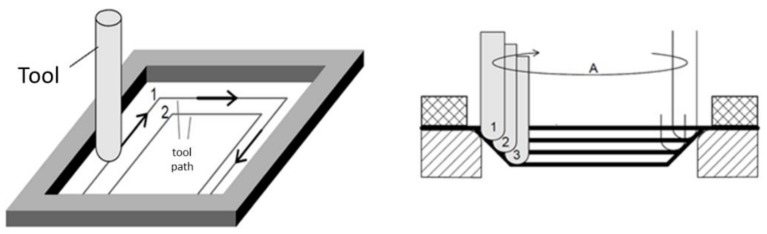
Schematic representation of the Single-Point Incremental Forming (SPIF) process.

In recent years, great attention has been paid to the low-cost manufacturing process known as Incremental Sheet Forming (ISF), which can be described in a general way as a process where a plastic deformation is applied locally and in a consecutive manner on a flat sheet until a final part with desired geometry is obtained [1]. One of the variations of ISF is the so-called Single-Point Incremental Forming process (SPIF), in which a simple-shaped tool moves horizontally and vertically by a toolpath program until the desired final part is formed [2] as depicted in Figure 1. The main motivation to further develop the SPIF process, and ISF in general, is the possibility to produce many different parts without the need to manufacture tooling, i.e., the toolpath defines the part geometry, so a new path can be programmed and used without incurring costs of tool development and switchover of setup [3]. For this reason, SPIF is considered as a manufacturing process with high economic payoff for rapid prototyping and small-batch production [4].

At its inception, SPIF emerged as a process used for the fabrication of sheet metal components [2,5,6,7]. However, around a decade ago, the use of SPIF was extended to fabricate workpieces from polymers, specifically thermoplastics [8,9,10,11], because of two main reasons: the structural and thermal properties of thermoplastics make them particularly suitable to applications in which a high strength/mass ratio and good formability are required, e.g., in the medical, aerospace, and automotive sectors. The second important reason is that SPIF enables the deformation of polymers at room temperature. This represents a significant cost saving because besides not requiring energy nor special equipment to melt the polymer, a mold is not needed either.

The research on SPIF of polymers has been directed towards the development of strategies for selecting the most appropriate polymers for SPIF [12], taking into account the ductility of the polymer and the required level of geometrical part accuracy, and for selecting the optimal parameters for the SPIF process. Some of the early work generated in this context was reported by Martins et al. [11], who made the first attempt towards the development of criteria for the selection of polymers that are suitable for SPIF by testing five different polymers: polyoxymethylene (POM), polyethylene (PE), polyamide (PA), polyvinylchloride (PVC), and polycarbonate (PC). In this work, the authors concluded that the feed rate, the thickness of the sheet, the diameter of the forming tool, and the step size play a key role in SPIF of polymers. In another work developed with PVC, Bagudanch et al. [13] concluded that the spindle speed has a significant influence on the temperature variation during the forming process and in the maximum force achieved, in such a way that the force needed to form polymers in SPIF is reduced as the temperature is increased.

Nevertheless, a lack of research has been detected regarding an in-depth study of the properties of polymers, mainly the thermal and structural ones, and their correlation with their performance in SPIF. For instance, the work reported by Martins et al. [11] studied the formability of the five thermoplastics previously mentioned based on their mechanical properties. Similarly, Marques et al. [4] based a study of the formability in SPIF of some polymers previously used, such as PVC, PC, and PA, and adding polyethylene terephthalate (PET) to this study, on a merely mechanical characterization and a qualitative consideration of the crystallinity using a classification from high-crystalline to amorphous. Davarpanah et al. [14] quantitatively analyzed, by using Differential Scanning Calorimetry (DSC), how different parameters varied in SPIF affect the crystallinity of polylactic acid (PLA). Lately, the use of characterization techniques based on X-ray have allowed for the observation that polymer chains are oriented as a consequence of the SPIF process. In a previous work, Lozano-Sanchez et al. [15] reported the orientation of polypropylene (PP) chains and suspected a difference in the behavior of polymer chains between the inner and outer surfaces of the analyzed sheets. This difference was related to different forces exerted on each surface, which was later supported by the work of Jiménez et al. [16] who found that the residual stresses were changing from tensile to compressive along the inner and outer surfaces of aluminum sheets processed by SPIF. Although this last work used metal sheets, it has been assumed that the same effect occurs on any material formed by SPIF, since this effect is attributed to the processing rather than to the material itself. Moreover, although it is widely accepted that the friction between the forming tool and the sheet generates enough heat to soften the polymer, there is no study that considers the thermal properties of polymers, since in thermoplastics the temperature plays a key role in its processing.

On the other hand, only few works related to the performance of biocompatible polymers in SPIF are found in the literature [14,17]. This represents an interesting area of opportunity considering that most of the materials tested up to now are non-biocompatible thermoplastics, and that one of the major advantages of SPIF is the possibility to manufacture customized products that can be applied in the medical field. Among the biocompatible polymers, polycaprolactone (PCL) is of great interest due to some of its properties, such as a low melting point (59–64 °C), exceptional blend-compatibility, high flexibility, and a medium Young’s modulus at room temperature, which has stimulated extensive research on its potential application in the biomedical field since 1980 [18]. Nowadays, the main commercial application of PCL is in the manufacture of biodegradable bottles and films, but according to Khan et al. [19], bio-medical applications, such as synthetic wound dressings, encapsulants for drug release systems, and contraceptive implants, are becoming increasingly common. Likewise, ultra-high molecular weight polyethylene (UHMWPE) has been widely used since the late 1960s in medical implants, such as total joint replacements in hips and knees [20,21], due to its biocompatibility coupled with its high strength and ductility resulting from the semi-crystalline structure of its long chains [22]. So, the use of these biocompatible polymers in SPIF have just gained interest in the last few years. For instance, PCL has lately been used for manufacturing cranial geometries [23,24,25], while both PCL and UHMWPE were included in a work in which was analyzed the influence of the main parameters, i.e., tool diameter, spindle speed, feed rate, and step down, in the maximum temperature reached during the SPIF process [26].

In this work, sheets of PCL and UHMWPE were fabricated by compression molding and then used in SPIF to fabricate pyramid-shaped parts with circular generatrix. The SPIF processing of both polymers was conducted using a Box–Behnken design of experiments varying four parameters: the diameter of the forming tool, the spindle speed, the feed rate, and the step down. The performance of these biocompatible polymers in SPIF was assessed through the formability, defined in terms of the maximum depth of the pyramid-shaped parts, the forming force, and the maximum temperature reached during the forming process. The PCL and UHMWPE sheets were characterized before and after being processed by SPIF in order to correlate their thermal and structural properties with the behavior displayed in SPIF. The results here presented seek to contribute to a better understanding of the behavior of polymers formed by SPIF in addition to encouraging the use of biocompatible polymers to achieve the development of this process aimed at one of its most interesting potential applications: the manufacture of custom medical implants.

## 2. Materials and Methods

### 2.1. PCL and UHMWPE Sheets Preparation

Fifty-two grams of PCL pellets (Sigma Aldrich, St. Louis, MO, USA, ≈3 mm, average *M*n = 80,000) were placed into the square cavity of a stainless steel mold with dimensions of 150 mm × 150 mm × 2 mm. The PCL pellets were compressed at 100 °C by using a CARVER 4122 hydraulic heating press (Carver Inc., Wabash, IN, USA), initially applying a load of 2 metric tons for 5 min to ensure the melting of the material. After that, the load was increased to 30 metric tons and maintained for 2 min. Finally, the load was removed and the mold was cooled to room temperature in a cooling press under a load of 30 metric tons as well. The fabrication of the UHMWPE sheets was carried out using the same procedure described before, using 46 g of UHMWPE powder (Sigma Aldrich, average *M*w: 3,000,000–6,000,000) compressed at 170 °C in the hydraulic heating press.

### 2.2. Materials Characterization

The PCL and UHMWPE sheets were studied before the SPIF processing by Vicat softening temperature (VST), differential scanning calorimetry (DSC), thermal gravimetric analysis (TGA), dynamic mechanical analysis (DMA), and tensile tests. The VST was determined by using a DTUL/Vicat tester model HDT-III (Custom Scientific Instruments, Easton, PA, USA). Tests were carried out with a total load of 50 N and a heating rate of 120 °C/h following the guidelines of the ASTM D-1525 standard. TGA and DSC analysis were carried out simultaneously in an SDT Q600 (TA Instruments, New Castle, DE, USA) with a precision of the thermobalance of 0.0001 mg. For this, small samples of about 20 mg were cut from the pre-fabricated sheets. Each sample was heated to 600 °C at 10 °C/min under an argon flow of 100 mL/min. DMA was carried out in a Dynamic Mechanical Thermal Analyzer model Q800 from TA Instruments used in the cantilever mode with a frequency of 1 Hz and amplitude of 20 μm. DMA was performed to determine the viscoelastic behavior of the materials through the measurement of the storage modulus (*E*’), loss modulus (*E*”), and loss factor (tan δ = *E*”/*E*’) over a temperature range of 30–70 °C for PCL, and 30–150 °C for UHMWPE, with a heating rate of 5 °C/min in both cases. The tensile tests were performed in a United universal testing machine equipped with a load cell of 4450 N, according to the ASTM 638 standard, using specimens of type 1. An extensometer was employed to measure the elongation of the specimens. The used rate was 50 mm/min for the analysis of the tensile strength and 5 mm/min for the modulus calculation. The initial separation between gags was 114 mm. The mechanical tensile properties of PCL and UHMWPE were taken as the average values obtained from a total of five specimens tested of each polymer. Some selected final parts fabricated by SPIF were analyzed by X-ray diffraction (XRD). The XRD analyzes were performed on a Empyrean diffractometer (PANalytical, Almelo, Netherlands) equipped with a Bragg–Brentano module and by using a X-ray tube source of Cu Ka radiation (λ = 1.5406 Å) operated at 40 kV and 45 mA. The diffraction patterns were obtained over the 2θ range of 5°–60° with steps of 0.02° and 50 s per step.

### 2.3. SPIF of PCL and UHMWPE Sheets

SPIF of PCL and UHMWPE sheets was conducted in a Kondia^®^HS1000 three-axis milling machine (Kondia, Spain). A fixed system consisting of a hollow support die was bolted over a dynamometer. The polymer sheets were placed between the clamping and the top plates with an effective working area of 120 mm × 120 mm. The incremental forming was made with a hemispherical Vanadis 23 steel tool. Around 5 mL of vegetable oil was spilled on the testing sheet in order to decrease friction effects between the forming tool and the testing material. The performance of the polymer sheets was evaluated through a Box–Behnken (BB) design of experiments considering four variables. BB designs are three-level designs that allow for fitting second-order response surfaces efficiently. The considered parameters and levels are listed in Table 1.

**Figure 2:**
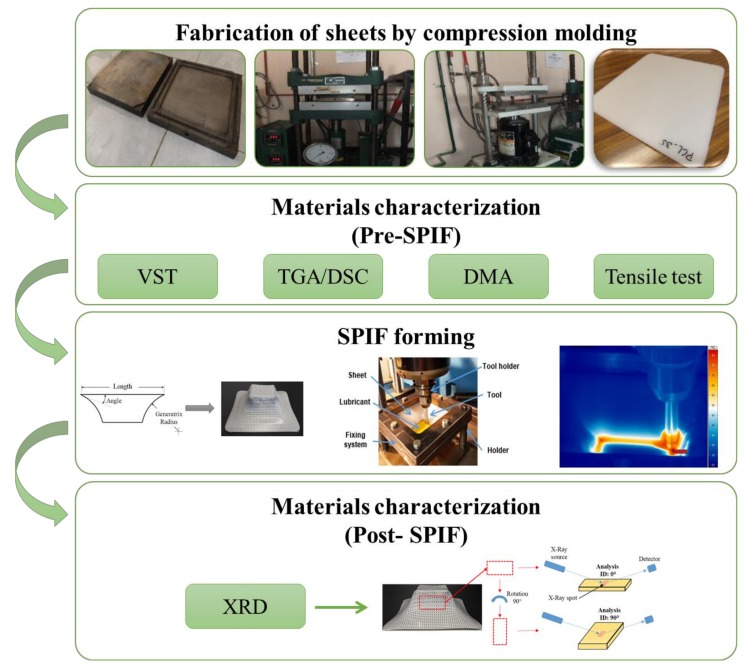
Summary of the experimental methodology.

The BB design with four factors consists of 27 experimental runs (Table 2) that can be split into three blocks with one center point at each block. During the SPIF processing, the force in the *z*-axis (*F*_z_) was measured by using a Kistler 9257B dynamometer and the temperature of the polymer sheets was measured with an Irbis ImageIR 3300 thermographic camera (InfraTec GmbH, Dresden, Germany). A summary of the experimental methodology used in this work is displayed in Figure 2.

## 3. Results and Discussion

### 3.1. Characterization of PCL and UHMWPE Sheets

**Figure 3:**
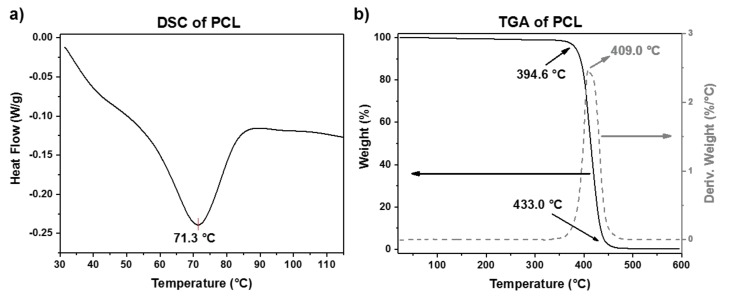
(**a**) DSC and (**b**) TGA plots of PCL.

The thermal properties of the PCL and UHMWPE sheets were studied by DSC, TGA, and Vicat test. From the Vicat test, the corresponding analysis determined that the Vicat softening temperature (VST) for PCL and UHMWPE is around 44 and 90 °C, respectively. The VST is considered as the temperature at which a specimen is penetrated to a depth of 1 mm by a flat-ended needle with a 1 mm^2^ circular or square cross-section. Is worth mentioning that the VST is a specific temperature value, while the melt point of thermoplastic polymers is usually taken within a whole range of temperature. So, for applications in SPIF, it will be more useful to consider the VST to analyze the formability of polymers once the sheet reaches the VST due to the friction caused by the contact between the sheet surface and the forming tool. The DSC and TGA curves for PCL are shown in Figure 3. The melting behavior of PCL displayed in Figure 3a shows a melting peak around 71 °C. From the TGA curve (Figure 3b), it can be observed that PCL shows a degradation step in the range from 394 to 433 °C, with a temperature of maximum decomposition rate of 409 °C. These results agree with the values reported in the literature [19,20].

**Figure 4:**
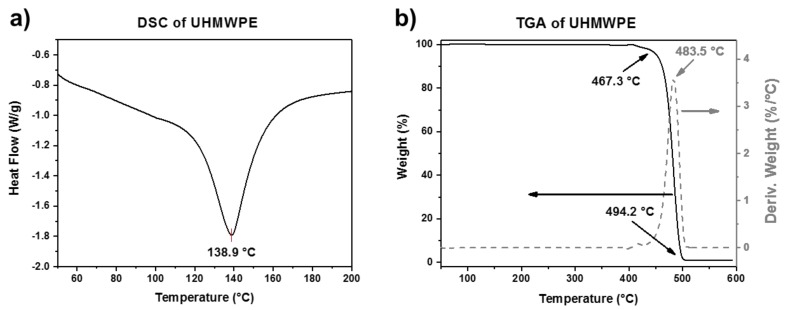
(**a**) DSC and (**b**) TGA plots of UHMWPE.

The DSC and TGA curves of UHMWPE are shown in Figure 4a,b, respectively. The DSC curve shows a melting peak at around 139 °C. On the other hand, the TGA curve also shows a single-step degradation behavior in the range from 467 to 494 °C, with a temperature of maximum decomposition rate around 484 °C. The higher thermal stability of UHMWPE compared to PCL is attributed to its longer polymer chains. The thermal properties of the two polymers are summarized in Table 3. 

**Figure 5:**
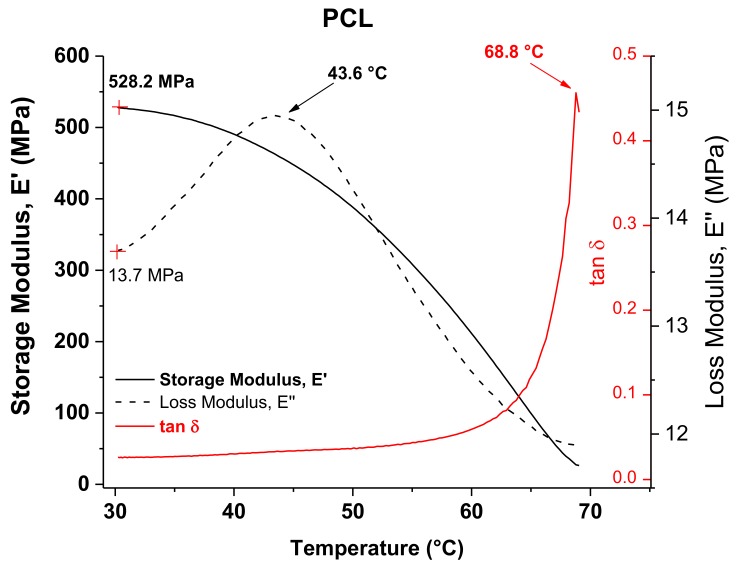
DMA graphs of PCL.

DMA results of PCL in the temperature range of 30–70 °C are shown in Figure 5 for the temperature dependence of the storage (*E*’) and loss (*E*”) moduli and the loss factor (tan δ). Observed at the initial temperature of the analysis were values of *E*’ and *E*” equal to 528 and 113.7 MPa, respectively. The curve of *E*” shows a peak that has been attributed to a transition occurring in the polymer associated with the slippage between crystallites. This type of transition has often been identified as an alpha star transition (*T*_α_*) and detected in semicrystalline polymers [27]. This peak has a maximum value at 43.6 °C, which is close to the VST previously determined for PCL. The alpha star transition was not detected by DSC because DMA is much more sensitive and can easily measure transitions not apparent in other thermal methods. This *T*_α_* can be correlated with the VST if it is considered that at a temperature around 44 °C there is a crystal–crystal slippage within the PCL, which facilitates the penetration of the needle in the VST test. The curve of tan δ shows a peak at 68.8 °C, which corresponds to the melting temperature (*T*_m_) and is similar to that previously measured by DSC.

**Figure 6:**
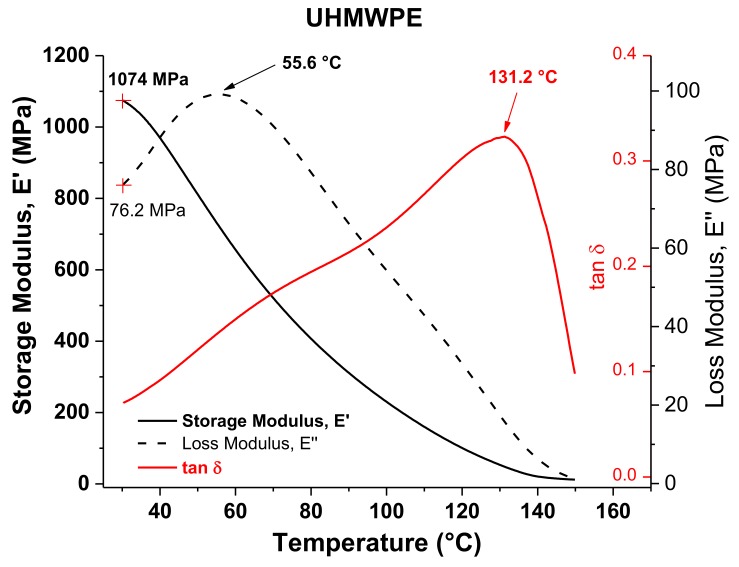
DMA graphs of UHMWPE.

Figure 6 shows the DMA results of UHMWPE. Similar to what occurs with PCL, the *E*” curve of UHMWPE exhibits a peak at 55.6 °C, which can also be identified as an alpha star transition associated with the slippage between crystallites, considering that it is a semicrystalline polymer as well. Unlike PCL, the values of VST and *T*_α_* of UHMWPE differ considerably from each other. This can be attributed to the presence of long molecular chains in UHMWPE, so that even when there is a crystal–crystal slippage at around 55 °C, it becomes more complicated to move or pass through a polymer composed of long chains that must also be highly entangled as occurs in UHMWPE. Therefore, it is necessary to continue heating to allow for sufficient movement of the long chains so that the VST test needle penetrates in the UHMWPE structure. The *T*_m_ obtained by DMA, taken as the peak of the tan δ curve (i.e., 131.2 °C), is similar to that obtained by DSC.

**Figure 7:**
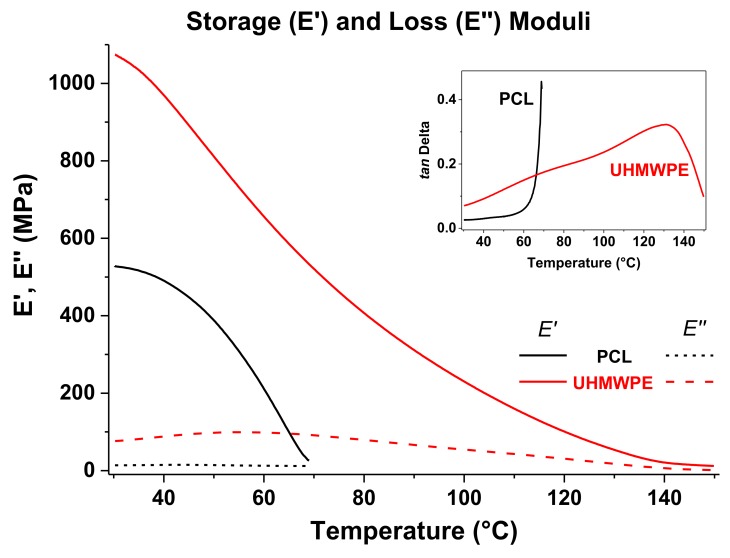
Storage and loss moduli from DMA for PCL and UHMWPE. The inset graph corresponds to the plots of tan δ.

The storage and loss moduli of PCL and UHMWPE have been plotted together in Figure 7, and the tan δ curves are shown in the inset graph. For both polymers, *E*′ reduced gradually as temperature increased, which is typical in thermoplastics and represents that less force is required for deformation; however, it is observed that *E*′ of PCL decreases more sharply and UHMWPE displays higher *E*’ values meaning a more rigid structure. In other words, based on the DMA results it would be more difficult to plastically deform UHMWPE than PCL. In the inset figure, the tan δ curve of PCL shows a pronounced increase as it approaches the *T*_m_, unlike the tan δ curve of UHMPE that exhibits a continuing increase throughout the whole temperature sweep. The temperature transitions in UHMWPE, specifically the melting transition, occur more gradually due to the presence of long molecular chains in a manner that before the melt is reached, i.e., where large-scale chain slippage occurs and the material flows, the coiled long chains must be disentangled first.

**Figure 8:**
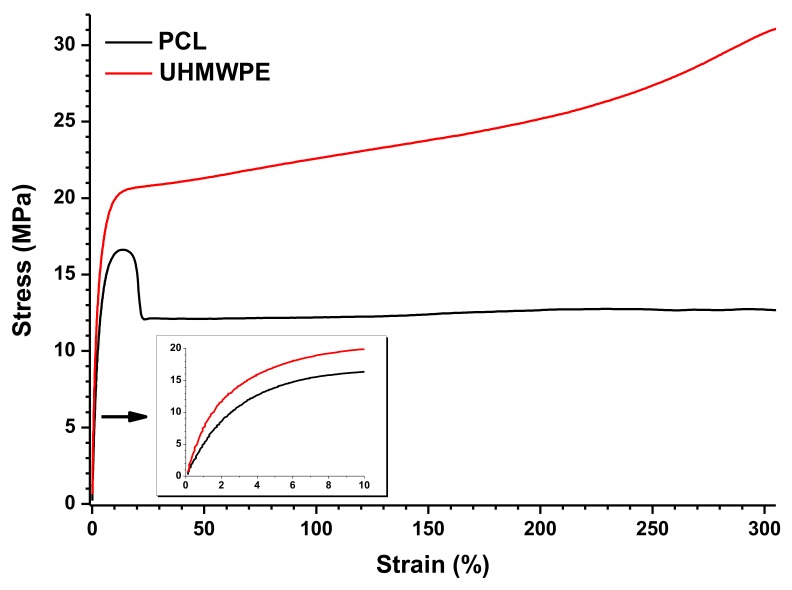
Tensile tests of PCL and UHMWPE.

The results from tensile tests of PCL and UHMWPE are graphically shown in Figure 8. For this, the graph of only one specimen of each material was taken, but it is properly representative of the behavior observed in all the specimens tested. During the tensile test, PCL shows a decrease in strength after the yield point, although it is maintained in a stable value. This behavior is typically observed when the specimen undergoes a necking effect. On the other hand, UHMWPE shows a continuous increase of the supported tensile stress after the yield point, which is clear evidence that a strain hardening effect occurs in the specimen. The inset graph in Figure 8 represents the initial stage of the stress versus strain curve, where a higher Young’s modulus is observed in UHMWPE indicating a more rigid material as was also observed by DMA. The mechanical properties from the tensile tests are summarized in Table 4. From these data, it was determined that the ultimate tensile strength of PCL and UHMWPE was 16.4 and 20.2 MPa, respectively. It is worth mentioning that the tensile strength of UHMWPE was taken at a point on the stress versus strain curve near where the plastic deformation begins, so the strain hardening effect is not considered (see Figure 8). Both materials showed a highly ductile behavior with an average elongation at the end of the test of more than 450% and 340% for PCL and UHMWPE, respectively. Moreover, due to the strain hardening effect observed in UHMWPE, it is clear that it has higher toughness than PCL.

### 3.2. SPIF of PCL and UHMWPE Sheets

The results obtained from the BB design of experiments were statistically analyzed by means of the response surface methodology; however, a behavior similar to that previously reported by Bagudanch et al. [26] was found even when the PCL and UHMWPE sheets corresponded to polymers with different properties to those used in this work. So, the detailed analysis of the response surface methodology can be reviewed in detail in [26]. In the present work, the effect that the parameters considered in the design of experiments have when processing PCL and UHMWPE by SPIF will be briefly analyzed by using box diagrams in order to make a correlation between the behavior shown in the forming process and the properties studied by the different characterization techniques used here.

**Figure 9:**
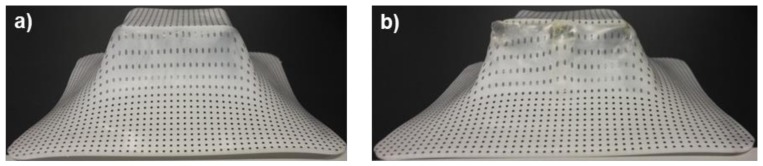
Pyramid-shaped parts made of PCL. (**a**) Sheet completely formed; (**b**) Sheet with failure.

The maximum formed depth (in percentage), forming force in the *z*-axis (*F*_z_), and maximum temperature reached (*T*_max_) in each test done from the BB design of experiments are summarized in Table 5 for the PCL sheets and in Table 6 for the UHMWPE sheets. For convenience, the conditions of each test (enlisted previously in Table 2) have been listed again. The results for PCL (see Table 5) show that most of the experiments reached a depth of 100%. The images in Figure 9 represent sheets of PCL completely formed and with failure. The pyramid-shaped part of Figure 9b corresponds to the sheet formed under parameters of experiment #15, which failed at a depth of 95%. This experiment is particularly interesting because it registered the maximum temperature (67.52 °C) among all of the tests made with PCL, which caused a very irregular surface as observed on the walls of the pyramid-like part. The temperature reached in this experiment is close to the *T*_m_ of PCL, so that at the end of the SPIF processing, the polymer likely behaved more as a viscous liquid than as a solid.

**Figure 10:**
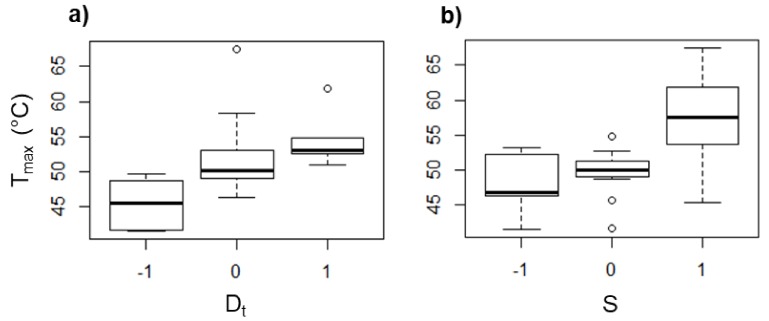
Box diagrams of *T*_max_ (in °C) for PCL as a function of (**a**) *D*_t_ and (**b**) *S*.

**Figure 11:**
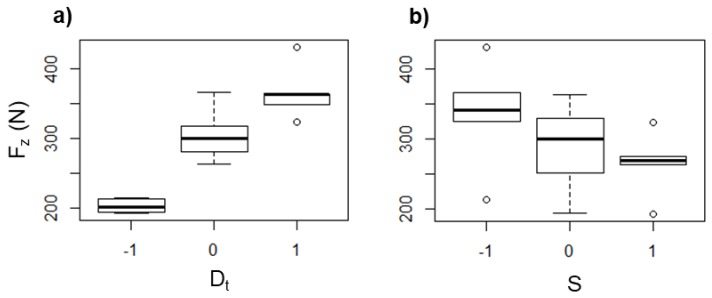
Box diagrams of *F*_z_ (in N) for PCL as a function of (**a**) *D*_t_ and (**b**) *S*.

From the design of experiments, it was found that *D*_t_ and *S* are the parameters that mostly affect *F*_z_ and *T*_max_. The box diagrams displayed in Figure 10 and Figure 11 show the tendency of *T*_max_ and *F*_z_, respectively, in PCL as an effect of *D*_t_ and *S*. The effect of the feed speed and the step size (data not shown) was found to be negligible for *T*_max_ and *F*_z_. It is noticed that *T*_max_ increases as *D*_t_ is increased (see Figure 10a). When a forming tool of larger diameter is used, the area in contact with the polymer sheet increases, generating a greater friction between both surfaces and consequently a higher temperature. A similar behavior associated with the friction occurs when *S* is varied (see Figure 10b), since there is more friction between surfaces when the tool rotates faster, i.e., the temperature increases when *S* is increased. In fact, it has been reported that the spindle speed has the most important role in the temperature variation in the SPIF of polymers [13].

On the other hand, *F*_z_ increased with larger *D*_t_ (see Figure 11a). This could be a result of the higher contact area between the forming tool and the polymer sheet, and with this, there is a greater amount of material that must be pushed down. Meanwhile, the force is reduced as *S* is increased (see Figure 11b), which can be directly associated with the softening of the polymer as a consequence of the temperature increase due to the friction between the tool and the polymer sheet as was mentioned before. In general, when the temperature is increased, the forming force is reduced because the polymer undergoes a softening.

**Figure 12:**
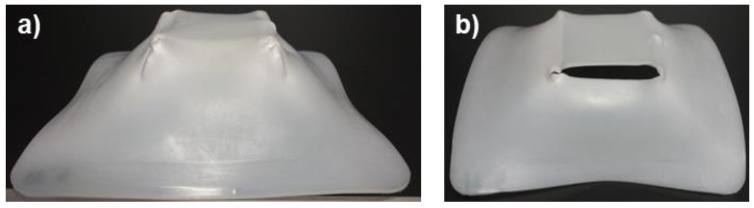
Pyramid-shaped parts made of UHMWPE (**a**) Sheet completely formed; (**b**) Sheet with failure.

For the case of UHMWPE, Table 6 shows that most of the sheets tested in SPIF formed to 100% of the final geometry; however, more sheets fractured compared to PCL. Figure 12 shows a pyramid-like part completely formed and and another with failure. At first sight, it is observed that the geometric precision of the pyramids is low since the shape of the manufactured parts has a curvature in the walls that is very different from that originally designed by the toolpath.

**Figure 13:**
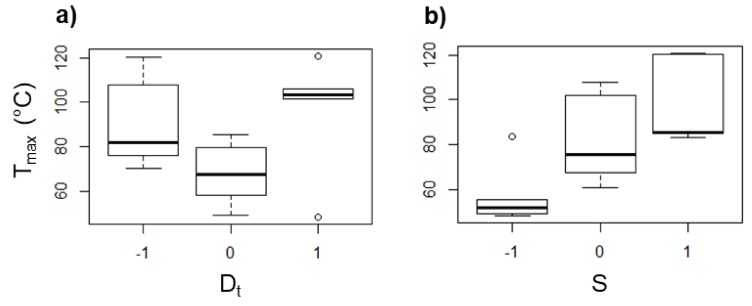
Box diagrams of *T*_max_ (in °C) for UHMWPE as a function of (**a**) *D*_t_ and (**b**) *S*.

An increase of *S* leads to a remarkable rise of the heat generated due to the tool–sheet friction as determined from the values of *T*_max_ measured with rotation of the tool. In this regard, in some experiments with UHMWPE sheets, *T*_max_ values of up to 120 °C were reached with *S* = 2000 rpm, while for some experiments without rotation, the *T*_max_ registered was below 50 °C. In general, the values of *T*_max_ measured for the UHMWPE sheets show more variation than that observed in the experiments with PCL, as it can be seen in the box diagrams of Figure 13, and additionally, there is not a clear tendency of *T*_max_ as a function of *D*_t_ (see Figure 13a), so it could be stated that the long chains of UHMWPE can generate a random behavior during the SPIF processing. Nonetheless, it can be still concluded that *T*_max_ increases as *S* increases (Figure 13b), which reaffirms that, regardless of the molecular structure of the polymer, *S* is the parameter in SPIF that most influences the temperature reached in the sheet.

**Figure 14:**
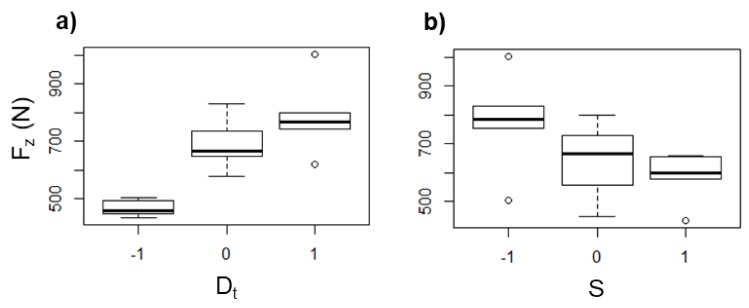
Box diagrams of *T*_max_ (in °C) for UHMWPE as a function of (**a**) *D*_t_ and (**b**) *S*.

The *F*_z_ in UHMWPE shows the same tendency previously observed in PCL as a function of *D*_t_ and *S*, i.e., *F*_z_ increases as *D*_t_ increases (Figure 14a) and decreases as *S* increases (Figure 14b), which is attributed to the same as discussed earlier for the case of PCL, i.e., a greater *D*_t_ represents a greater amount of material pushed down, and so the *F*_z_ is higher, while a higher *S* generates more heat due the friction between the tool and the sheet, which in turn softens the polymer, and consequently *F*_z_ is reduced.

### 3.3. Characterization by XRD after the SPIF Processing

Pyramid-shaped parts of PCL and UHMWPE formed by SPIF were analyzed by XRD in order to assess the molecular chain orientation as a result of the SPIF processing. In a previous work, Lozano-Sánchez et al. [15] observed by small- and wide-angle X-ray scattering (SWAXS) the orientation of Polypropylene chains in the vertical direction of cone-shaped parts as a result of SPIF processing. Nonetheless, it should be pointed out that the SWAXS analysis is completed through-thickness of the sample. Here, XRD was used to separately analyze the inner and outer surfaces of the wall of the pyramid-shaped parts based on the fact that compressive and tensile stresses are exerted on each side of sheets formed by SPIF as was previously concluded by Jiménez et al. [16]. Due to the large amount of samples formed from the design of experiments, the XRD analysis was completed in the pyramid-like parts obtained from only four different experiments: 9, 10, 11, and 12, which vary *D*_t_ and ∆*z* from the lowest value to the highest (*D*_t_ = 6, 14 mm; ∆*z* = 0.2, 0.5 mm), and the spindle speed and feed rate remained unchanged and in the middle value (see Table 2). Only variations of *D*_t_ and ∆*z* were considered since these parameters directly influence the shaping of the polymer in the vertical direction, i.e., the direction of descent of the forming tool, during SPIF processing.

**Figure 15:**
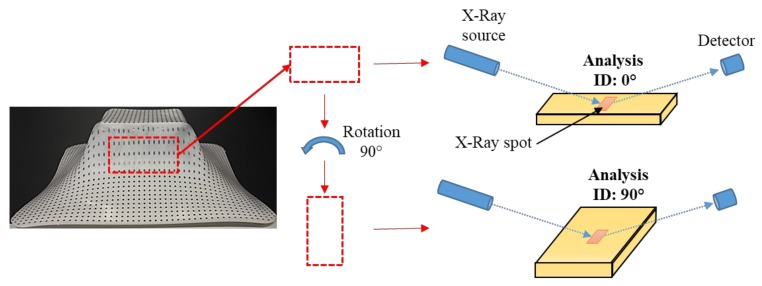
Configuration of the XRD analysis on a rectangular sample taken from the wall of the pyramid-shaped parts. The analysis were identified as “0°” and “90°” after rotation of the analyzed specimen as illustrated.

For the XRD analysis, a rectangular sample was taken from the wall of the pyramid-shaped parts. All of the samples analyzed correspond to the side opposite to the descent side of the forming tool. The samples were first analyzed in one direction with respect to the X-ray incident beam, and then a second analysis was completed after rotating the sample 90° as is schematically illustrated in Figure 15. This rotation of the samples was done in order to detect differences in the intensity of the diffraction peaks associated with the crystallographic planes of the polymer unit cell, mainly the {hk0} plane groups, that is, those that are parallel to the *c*-axis. If the molecular chains of the polymer are preferably oriented in one direction, a greater intensity could be seen in the diffraction peaks since the planes would also be elongated in the longitudinal axis of the oriented polymer chains, i.e., along the *c*-axis. Here, it should be emphasized that *x*, *y*, *z* coordinates are used as reference directions in the pyramid-shaped parts, where *z* corresponds to the vertical direction or the direction of descent of the forming tool, being *x* and *y* the directions of the horizontal plane, while the *a*, *b*, *c* coordinates are used as reference directions for the crystal unit cell for both PCL and UHMWPE crystalline structures.

**Figure 16:**
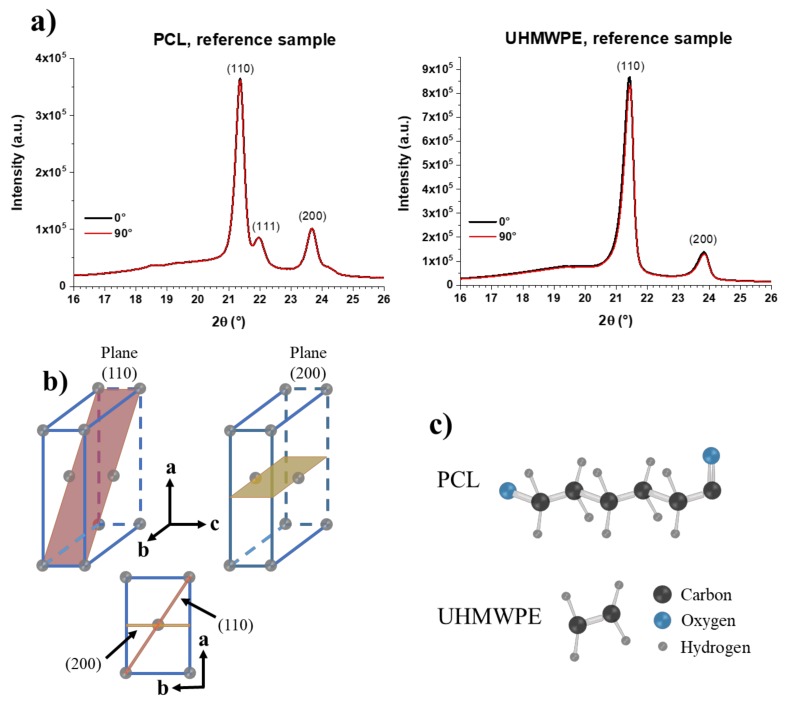
(**a**) XRD patterns of PCL and UHMWPE reference samples, i.e., unformed sheets; (**b**) Schematic representation of planes (110) and (200) of an orthorhombic crystal cell; (**c**) Repeat units of PCL and UHMPWE. A repetitive unit is located in each of the gray points that form the orthorhombic unit cell in (**b**).

First, the XRD patterns of the PCL and UHMWPE reference samples, i.e., unformed sheets, are shown in Figure 16a. These samples were analyzed in only one side considering that they are unformed, so the existence of inner and outer surfaces does not apply. In fact, it is expected that there is no difference in the order of the molecular chains on both sides of the unformed sheets. The XRD patterns show peaks of the diffraction planes (110) and (200) of the orthorhombic unit cell in around 21.4° and 23.7°, respectively, for both PCL and UHMWPE. The pattern of PCL shows a small peak at 22° associated with planes (111), which are also of the orthorhombic unit cell [28]. From these XRD patterns, it is concluded that both polymers have a semi-crystalline structure, evidenced by the well-defined, high-intensity peaks observed, which correspond to the crystalline part, and to the wide, low-intensity signal that can be seen at 2θ angles below 21°, which corresponds to the amorphous part. As was expected, the XRD patterns of the unformed sheets of PCL and UHMWPE show no difference between the measurements made at 0° and 90°, indicating that there is no chain orientation in these samples. The diffraction planes (110) and (200) of an orthorhombic unit cell are schematically represented in Figure 16b. It should be noted that the polymer chains extend along the *c*-axis. The structures of the repeat unit of PCL and UHMWPE are shown in Figure 16c. A repeat unit corresponds to each of the gray spheres that make up the orthorhombic cell shown in Figure 16b.

**Figure 17:**
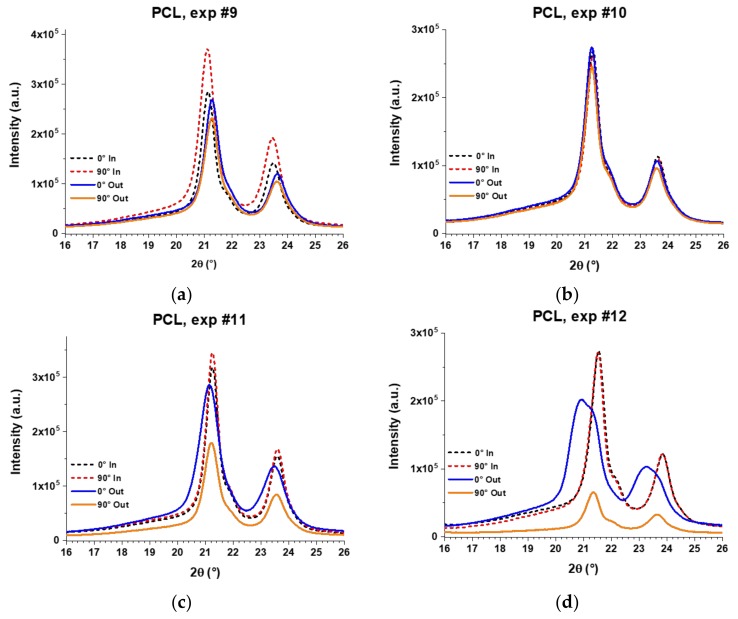
XRD patterns of PCL sheets formed by SPIF through experiments (**a**) #9; (**b**) #10; (**c**) #11; and (**d**) #12. The XRD analysis was carried out separately for the inner surface (in contact with the forming tool) and the outer surface (not in contact with the forming tool) according to the experimental setup described in Figure 15.

The XRD patterns of the PCL final parts fabricated through the experiments 9, 10, 11, and 12 are shown in Figure 17. The dotted lines correspond to the measurements made on the inner surface and the solid lines to those made on the outer surface of the wall of the pyramid-shaped parts. The XRD patterns of formed parts still show the peaks of (110) and (200) planes at around 21.4° and 23.7°, respectively. However, the peak attributed to the (111) plane is rather observed as an overlapped shoulder with the (110) peak, which evidenced a reduced crystallinity in the formed samples compared to the unformed sheet (i.e., reference PCL, see Figure 16a). In fact, some peaks show a clear broadening that also proves a reduction in crystallinity. In addition, it is possible to observe a clear shift of the diffraction peaks in the XRD patterns of the PCL formed sheets, which is due to the inherent curvature in the final parts resulting from the forming process. For a structural comparison between the analyzed samples, this shift will be dismissed and attention will be paid only to the maximum intensities and width of the peaks.

Furthermore, the XRD patterns in Figure 17 demonstrate a different molecular behavior between the inner and outer surfaces, except in experiment 10 where diffraction patterns are practically equal. This different behavior is more evident in experiments 11 and 12, where patterns of the outer surface are wider than those of the inner surface. In fact, in “Exp. #12”, the broad peaks obtained on the outer surface show a crystallinity that is well below the crystallinity observed on the inner surface. Interestingly, on the outer surface, the patterns obtained at 0° are more intense than those obtained at 90°, proving that PCL molecular chains are oriented in the vertical direction of the pyramid-shaped parts, that is, the direction of descent of the forming tool, according to the experimental setup used in this work for the XRD analysis (see Figure 15). On the other hand, the XRD patterns obtained on the inner surface show more intense peaks at 90° than at 0°, especially in experiments 9 and 11, indicating that in the inner surface, the PCL chains are preferentially oriented in the horizontal direction. These results suggest that PCL molecular chains are oriented horizontally in the inner surface due to the action of the forming tool, because it moves almost completely along the *x* and *y* directions (except for the small region where the tool steps down) and in this movement it could be “pulling” the molecular chains in the same direction. The difference between the patterns obtained at 0° and 90° on the inner surface is small, perhaps due to the fact that the movement of the forming tool is alternating, that is, a bidirectional contouring, which would be inhibiting a greater chain orientation.

In the outer surface, the polymer chains are not in contact with the forming tool, but these are incrementally stretched along the vertical direction at the same time and in the same way as the pyramid-shaped part is incrementally formed. As was mentioned before, the orientation of polymer chains in the vertical direction was observed by means of SWAXS in polypropylene sheets [15]; however, in those previous results, the horizontal orientation of chains in the inner surface could not be observed because the SWAXS analysis is performed through-thickness of the sheet. Nonetheless, in that previous work the authors obtained a hint of the difference in molecular behavior of the polymer between the inner and outer surfaces when they noticed a whitening on the polypropylene sheets after the SPIF processing and highlighted that this whitening only occurred on the outer surface of the formed parts. This whitening has also been observed in PVC [4], and is attributed to a crazing effect, which, according to McLeish et al. [29], is related to the disentanglement of chains.

**Figure 18:**
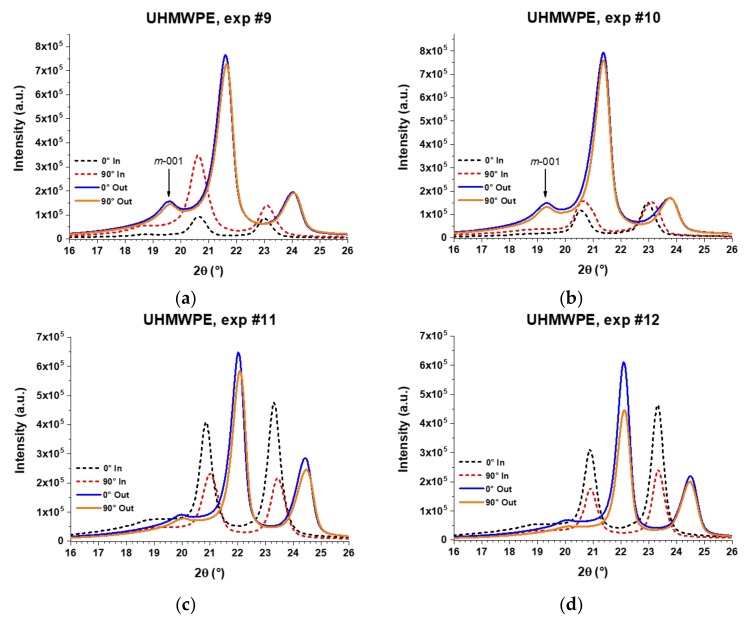
XRD patterns of UHMWPE sheets formed by SPIF through experiments (**a**) #9; (**b**) #10; (**c**) #11; and (**d**) #12. The XRD analysis was carried out separately for the inner surface (in contact with the forming tool) and the outer surface (not in contact with the forming tool) according to the experimental setup described in Figure 15.

Figure 18 shows the XRD patterns in the 2θ range from 16° to 26° of the pyramid-shaped parts of UHMWPE. The shift of peaks is more evident than in the case of the PCL sheets because the curvature of the UHMWPE parts is greater than in the parts of PCL, as can be observed in the pyramid parts shown in the images of Figure 9 and Figure 12. All patterns of the UHMWPE formed sheets show the peaks of (110) and (200) planes, and it is evident that both surfaces, inner and outer, behave differently when they are processed by SPIF. Regarding the inner surface, in experiments 9 and 10, the patters obtained at 90° are more intense than those at 0°, suggesting that polymer chains are oriented horizontally, similar to what happens with PCL. However, in experiments 11 and 12, the opposite occurs, i.e., the peaks at 0° are more intense, indicating that in the formed parts fabricated through these experiments, the polymer chains are preferentially oriented along the vertical direction. Experiments 11 and 12 were performed with the larger *D*_t_, which should contribute to this difference in chain orientation.

**Figure 19:**
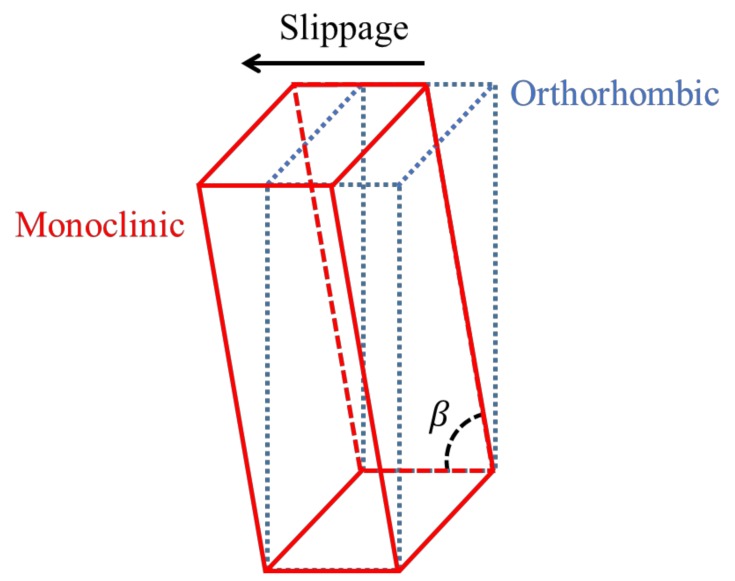
Schematic representation of the distortion from the orthorhombic unit cell to a monoclinic unit cell.

Regarding the outer surface, the XRD patterns of UHMPWE formed parts show a slight difference in intensities between the measurements obtained at 0° and 90° in experiments 9, 10, and 11, but in experiment 12, the difference in intensities is more marked, being greater for the measurement carried out at 0°, which indicates that the polymer chains are oriented in the vertical direction. It is worth noting that in experiments 9 and 10, the patterns obtained on the outer surface show a peak in the 2θ range of 19.3°–19.7°, which is associated to the (001) diffraction plane of the monoclinic structure of UHMWPE [30]. Thus, it is possible to infer that the movement of chains in a preferential direction generates a distortion in the initial orthorhombic unit cell. In fact, a monoclinic unit cell differs from an orthorhombic in only one of the angles between planes, specifically the angle β between the cell parameters *a* and *c*, being β ≠ 90° in the monoclinic unit cell*,* and β = 90° in the orthorhombic unit cell. This distortion in the unit cell, which is schematically represented in Figure 19, can be related to the slippage between crystallites that was previously detected by DMA and identified as the alpha star transition. Such distortion of the unit cell was not observed in PCL, probably due to the fact that the crystalline region of the UHMWPE is more compact because it contains a linear structure with carbon-carbon bonds in the main chain while the structure of PCL contains carbonyl groups (see Figure 16c).

The chain orientation in the outer surface of the UHMWPE formed parts was less than that observed in the PCL ones because of the larger size of the UHMWPE chains. The forming tool generates the movement of chains on the surface of the sheet that is in direct contact with the tool (i.e., the inner surface). This produces a rearrangement of the polymer chains that continues along the thickness of the sheet, that is, towards the outer surface. However, this rearrangement requires the movement of the long, coiled chains of UHMWPE, which is hardly carried out. In fact, the DMA results showed that in UHMWPE, more energy is lost due to friction and internal movements compared to PCL (the loss modulus, *E*”, of UHMWPE is higher); however, the tan δ curve of UHMWPE showed a gradual increase as the temperature is also increased, while that of PCL increased more abruptly. Considering that tan δ is a measure of how efficiently a material loses energy due to molecular rearrangements, these results indicate that in the case of UHMWPE, the lost energy is scarcely used for molecular rearrangement, while in PCL the opposite occurs, mainly as it approaches the melting temperature where the energy is efficiently harnessed for molecular motion and the consequent flow of the material.

**Figure 20:**
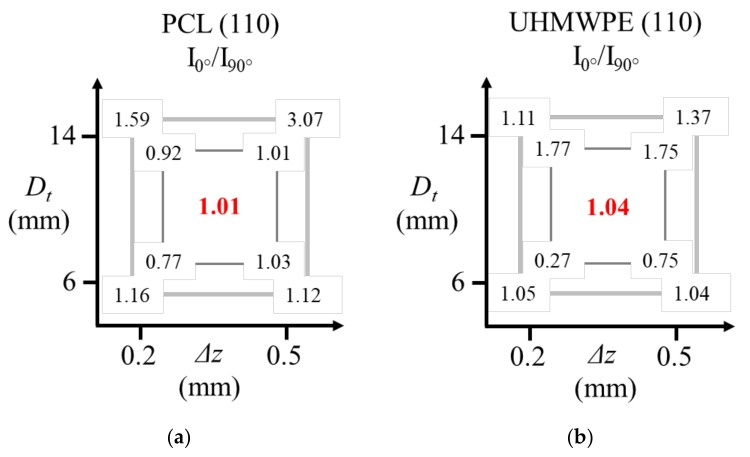
Values of the ratio *I*_0_/*I*_90_ for the diffraction peak associated to the (110) plane (**a**) PCL; (**b**) UHMWPE; where *I*_0_ is the maximum intensity in the measurement at 0° and *I*_90_ is the maximum intensity in the measurement at 90° according to the experimental setup described in Figure 15.

In order to analyze how the parameters *D*_t_ and ∆*z* affect the chain orientation in the formed parts, Figure 20 presents the values of the ratio *I*_0_/*I*_90_ for the diffraction peak associated to the (110) plane, where *I*_0_ corresponds to the maximum intensity obtained in the measurement at 0° and *I*_90_ corresponds to the maximum intensity in the measurement at 90°. In this way, a value of *I*_0_/*I*_90_ = 1 represents that the polymer chains are not oriented in a preferential direction, a value of *I*_0_/*I*_90_ >1 indicates that the chains are oriented vertically in the pyramid-shaped parts, and *I*_0_/*I*_90_ <1 indicates that the chains are oriented horizontally. If the value of *I*_0_/*I*_90_ is farther from 1, it means that the chain orientation is greater. The graphs show the results for the inner surface (small internal square) and for the outer surface (large external square). The central red values represent the *I*_0_/*I*_90_ ratio of the corresponding reference sample (unformed sheet) of each polymer. This value for PCL and UHMWPE is close to 1, since the polymer chains are not oriented in these unformed sheets as was mentioned before.

As has been discussed, in the formed parts of PCL, the molecular chains are preferentially oriented in the horizontal direction in the inner surface. The experiment performed with *D*_t_ = 6 mm and ∆*z* = 0.2 mm generates the greater orientation of chains in the inner surface. One can think that a small tool concentrates the pulling of chains in a smaller region, and thus the crystalline parts of the polymer are oriented in the feed direction of the tool. A larger *D*_t_ implies a less-concentrated molecular movement, so that it could be causing the movement of a large amount of crystalline parts, but also of a large amount of the amorphous part of the polymer. To some extent, a small Δ*z* also represents a way of concentrating the movement of chains in a small size region. If the step size is small, in a second step down, the chains of the previous step can still be pulled by the forming tool, something that would not happen if the step size were larger, where the chains that were pulled in a previous step would hardly be affected by the displacement of the tool in the subsequent steps. Based on the aforementioned, a smaller Δ*z* causes a higher chain orientation, although this parameter is not as significant as *D*_t_ is for the orientation of chains in the inner surface.

Concerning the outer surface, *D*_t_ causes the most marked impact in the orientation of chains, which could be attributed to the same reason explained before regarding the large amount of material that is pulled by the forming tool when its diameter is larger. However, in the outer surface the orientation is preferentially towards the vertical direction of the pyramid-shaped parts because on this surface, the tool path in the *x-y* plane does not have an impact. Therefore, regardless of the direction, the orientation of polymer chains in the outer surface is higher than in the inner surface because in the outer surface the polymer is pulled only in one direction, that is, downwards.

In the case of UHMWPE, the parameter that most influences the orientation is *D*_t_. For a polymer that contains very long chains, such as UHMWPE, a tool of greater diameter creates a greater contact area between the tool and the surface of the sheet, which can be a more significant parameter compared to a polymer whose chains are not that big (the case of PCL). On the inner surface, a small *D*_t_ generates the horizontal orientation, while the larger *D*_t_ gives rise to the vertical orientation. The orientation of the chains on the outer surface is less evident than in the case of the PCL due to the energy lost by the molecular rearrangement from the inner surface to the outer one. Only a combination of parameters consisting of the largest *D*_t_ and the largest Δ*z* is able to generate a greater orientation on the outer surface because one could think that in this way a greater amount of material is pulled down and so a greater molecular rearrangement among the UHMWPE chains takes place.

The characterization of PCL and UHMWPE performed in this work before and after being processed by SPIF has revealed important thermal and structural properties that certainly should be considered when forming thermoplastics by SPIF. Even though the XRD analyzes were imprecise due to the curvature of the samples evaluated, this characterization technique allowed us to separately study the surface of the sheet that is in contact with the forming tool and the opposite surface that is not, and in this manner, it was clearly revealed that polymer chains behave differently on each surface. It is strongly believed that the use of techniques such as DMA and XRD may become a very useful tool for SPIF of polymers and that the results presented here can be extended to other semicrystalline thermoplastics.

## 4. Conclusions

In summary, sheets of the biocompatible polymers PCL and UHMWPE were fabricated by compression molding and shaped by SPIF with pyramid-like geometry through a BB design of experiments in which four parameters were varied. The performance of PCL and UHMWPE in SPIF was evaluated in terms of the maximum depth, *F*_z_, and *T*_max_ reached during the SPIF processing. The results indicated that *F*_z_, and *T*_max_ are mostly affected by the tool diameter and the spindle speed.

The thermal and structural properties of the polymers used were thoroughly studied. DMA revealed the existence of an alpha star transition (*T*_α_*) occurring in both polymers. During SPIF processing, it is key to reach the temperature of this transition for the forming of PCL and UHMWPE since it is associated with slippage between crystallites. XRD results proved that the polymer chains are preferentially oriented in different directions in the inner and outer surfaces of the pyramid-shaped parts. In the inner surface, the tool path contributes to the horizontal orientation of chains, while in the outer surface, the polymer chains are mainly pulled downwards, generating the vertical orientation of chains. The slippage of crystallites as a consequence of the forming process induces the distortion of the unit cell of UHMWPE, passing from orthorhombic to monoclinic.

It has been demonstrated that the characterization techniques used in this work are very useful in SPIF of polymers based on the thermal and structural information that is presented here for the first time. For instance, the use of XRD allowed the structural study of inner and outer surfaces separately. On the other hand, DMA supplied information about thermal transitions not readily identifiable by other methods, which makes it a very powerful tool in SPIF of polymers since the temperature is critical when forming these materials, especially thermoplastics.

## Figures and Tables

**Table 1:** Parameters and levels considered in the Box–Behnken (BB) design for SPIF of PCL and UHMWPE sheets.

Parameter	−1	0	1
*D*_t_ (tool diameter, mm)	6	10	14
*S* (spindle speed, rpm)	0	1000	2000
*F* (feed rate, mm/min)	1500	2250	3000
∆*z* (Step down, mm)	0.2	0.35	0.5

**Table 2:** List of 27 experiments for SPIF of PCL and UHMWPE sheets.

Exp.	Tool Diameter, *D*_t_ (mm)	Spindle Speed, *S* (rpm)	Feed Rate, *F* (mm/min)	Step down, Δ*z* (mm)
1	6	0	2250	0.35
2	6	2000	2250	0.35
3	14	0	2250	0.35
4	14	2000	2250	0.35
5	10	1000	1500	0.20
6	10	1000	1500	0.50
7	10	1000	3000	0.20
8	10	1000	3000	0.50
9	6	1000	2250	0.20
10	6	1000	2250	0.50
11	14	1000	2250	0.20
12	14	1000	2250	0.50
13	10	0	1500	0.35
14	10	0	3000	0.35
15	10	2000	1500	0.35
16	10	2000	3000	0.35
17	6	1000	1500	0.35
18	6	1000	3000	0.35
19	14	1000	1500	0.35
20	14	1000	3000	0.35
21	10	0	2250	0.20
22	10	0	2250	0.50
23	10	2000	2250	0.20
24	10	2000	2250	0.50
25	10	1000	2250	0.35
26	10	1000	2250	0.35
27	10	1000	2250	0.35

**Table 3:** Thermal properties of PCL and UHMWPE.

Sample	VST (°C)	*T*_m_ (°C)	Temp. of Initial Decomposition (°C)	Temp. of Maximum Decomposition Rate (°C)
PCL	44.3	71.3	394.6	409.0
UHMWPE	89.7	138.9	467.3	483.5

**Table 4:** Mechanical properties of PCL and UHMWPE from uniaxial tensile tests.

Sample	Young’s Modulus (MPa)	Yield Strength (MPa)	Elongation (%)
PCL	374.7	16.4	451.2
UHMWPE	445.3	20.2	343.0

**Table 5:** SPIF results of PCL sheets.

Exp.	*D*_t_ (mm)	*S* (rpm)	*F* (mm/min)	Δ*z* (mm)	Depth (%)	*F*_z_ (N)	*T*_max_ (°C)
1	6	0	2250	0.35	100	213.31	41.50
2	6	2000	2250	0.35	100	431.23	53.23
3	14	0	2250	0.35	100	192.02	45.32
4	14	2000	2250	0.35	100	323.42	61.90
5	10	1000	1500	0.20	100	308.92	50.91
6	10	1000	1500	0.50	100	299.78	49.23
7	10	1000	3000	0.20	100	298.03	49.13
8	10	1000	3000	0.50	100	309.45	49.11
9	6	1000	2250	0.20	100	301.49	50.16
10	6	1000	2250	0.50	100	193.24	45.76
11	14	1000	2250	0.20	100	362.43	50.90
12	14	1000	2250	0.50	100	196.85	49.67
13	10	0	1500	0.35	100	362.68	54.84
14	10	0	3000	0.35	100	366.14	52.25
15	10	2000	1500	0.35	95.08	262.67	67.52
16	10	2000	3000	0.35	100	324.69	47.13
17	6	1000	1500	0.35	100	262.24	58.27
18	6	1000	3000	0.35	100	287.48	50.04
19	14	1000	1500	0.35	100	214.77	48.69
20	14	1000	3000	0.35	100	348.03	52.77
21	10	0	2250	0.20	100	203.74	41.69
22	10	0	2250	0.50	100	362.48	52.58
23	10	2000	2250	0.20	100	354.03	64.50
24	10	2000	2250	0.50	100	274.20	53.75
25	10	1000	2250	0.35	100	327.08	46.35
26	10	1000	2250	0.35	100	273.95	56.94
27	10	1000	2250	0.35	100	291.83	51.56

**Table 6:** SPIF results of UHMWPE sheets.

Exp.	*D*_t_ (mm)	*S* (rpm)	*F* (mm/min)	Δ*z* (mm)	Depth (%)	*F*_z_ (N)	*T*_max_ (°C)
1	6	0	2250	0.35	96.72	503.16	83.82
2	6	2000	2250	0.35	96.72	433.23	120.36
3	14	0	2250	0.35	100	1003.60	48.24
4	14	2000	2250	0.35	100	619.47	120.85
5	10	1000	1500	0.20	100	685.67	60.89
6	10	1000	1500	0.50	100	617.41	67.63
7	10	1000	3000	0.20	100	713.51	75.75
8	10	1000	3000	0.50	100	641.53	75.03
9	6	1000	2250	0.20	100	494.03	107.84
10	6	1000	2250	0.50	100	452.33	70.01
11	14	1000	2250	0.20	100	799.40	102.14
12	14	1000	2250	0.50	100	785.14	104.01
13	10	0	1500	0.35	100	831.28	48.95
14	10	0	3000	0.35	100	754.38	52.71
15	10	2000	1500	0.35	83.61	658.87	83.06
16	10	2000	3000	0.35	84.43	577.47	85.39
17	6	1000	1500	0.35	100	466.49	79.48
18	6	1000	3000	0.35	100	449.15	75.89
19	14	1000	1500	0.35	100	742.55	101.54
20	14	1000	3000	0.35	100	745.89	105.94
21	10	0	2250	0.20	100	782.94	55.55
22	10	0	2250	0.50	79.07	580.94	84.80
23	10	2000	2250	0.20	100	788.07	51.23
24	10	2000	2250	0.50	100	653.74	85.46
25	10	1000	2250	0.35	100	665.85	67.52
26	10	1000	2250	0.35	100	660.19	65.57
27	10	1000	2250	0.35	100	699.30	62.69
